# Pharmacological Blockade of TRPM8 Ion Channels Alters Cold and Cold Pain Responses in Mice

**DOI:** 10.1371/journal.pone.0025894

**Published:** 2011-09-30

**Authors:** Wendy M. Knowlton, Richard L. Daniels, Radhika Palkar, Daniel D. McCoy, David D. McKemy

**Affiliations:** 1 Neurobiology Section, Department of Biological Sciences, University of Southern California, Los Angeles, California, United States of America; 2 Molecular and Computational Biology Section, Department of Biological Sciences, University of Southern California, Los Angeles, California, United States of America; 3 Neuroscience Graduate Program, University of Southern California, Los Angeles, California, United States of America; Georgia State University, United States of America

## Abstract

TRPM8 (Transient Receptor Potential Melastatin-8) is a cold- and menthol-gated ion channel necessary for the detection of cold temperatures in the mammalian peripheral nervous system. Functioning TRPM8 channels are required for behavioral responses to innocuous cool, noxious cold, injury-evoked cold hypersensitivity, cooling-mediated analgesia, and thermoregulation. Because of these various roles, the ability to pharmacologically manipulate TRPM8 function to alter the excitability of cold-sensing neurons may have broad impact clinically. Here we examined a novel compound, PBMC (1-phenylethyl-4-(benzyloxy)-3-methoxybenzyl(2-aminoethyl)carbamate) which robustly and selectively inhibited TRPM8 channels *in vitro* with sub-nanomolar affinity, as determined by calcium microfluorimetry and electrophysiology. The actions of PBMC were selective for TRPM8, with no functional effects observed for the sensory ion channels TRPV1 and TRPA1. PBMC altered TRPM8 gating by shifting the voltage-dependence of menthol-evoked currents towards positive membrane potentials. When administered systemically to mice, PBMC treatment produced a dose-dependent hypothermia in wildtype animals while TRPM8-knockout mice remained unaffected. This hypothermic response was reduced at lower doses, whereas responses to evaporative cooling were still significantly attenuated. Lastly, systemic PBMC also diminished cold hypersensitivity in inflammatory and nerve-injury pain models, but was ineffective against oxaliplatin-induced neuropathic cold hypersensitivity, despite our findings that TRPM8 is required for the cold-related symptoms of this pathology. Thus PBMC is an attractive compound that serves as a template for the formulation of highly specific and potent TRPM8 antagonists that will have utility both *in vitro* and *in vivo*.

## Introduction

The cold and menthol-gated ion channel TRPM8 [1], [2] serves as a neuronal sensor of cold temperatures and is essential for innocuous cool and noxious cold sensations [3], [4], [5], [6], [7]. Mice lacking functional TRPM8 channels are unable to discriminate between mildly warm and mildly cool temperatures, and do not show normal aversion to temperatures in the noxious cold range [6]. Moreover, recent evidence suggests that the channel is necessary for increased cold sensitivity associated with injury [4], [8], [9]. Somewhat paradoxically, the channel is also required for the analgesia (pain relief) associated with mild cooling and cooling compounds [5], [10]. Additionally, TRPM8 has recently been reported to be involved in thermoregulation [11], a role that is not entirely unexpected given that other temperature sensitive ion channels, particularly the heat-gated TRPV1, have also been implicated in regulating body temperature [12]. For example, several studies have shown that TRPV1-null mice display attenuated fever responses, and TRPV1 antagonism induces thermogenesis in rats and humans [12], [13], [14], [15].

Because TRPM8 is involved in a broad and diverse range of physiological processes, particularly those relevant to human health and disease, pharmaceuticals that manipulate channel function are needed both as tools for further study of the channel, and as potential therapeutic compounds [16]. With regard to agonists, at least eighteen natural and synthetic compounds have been found to activate the channel, including menthol, icilin, and eucalyptol [1], [2], [16]. While many agonists have been described, there are few channel antagonists reported in the literature, and none that are selective for TRPM8 [16]. Of those reported, many also act on other somatosensory-related ion channels, such as TRPV1 and the irritant receptor TRPA1, suggesting a conserved mechanism amongst these channels [17], [18], [19]. Capsazepine, a well-known TRPV1 antagonist, also has non-specific activity on voltage-gated calcium channels, nicotinic acetylcholine receptors, and TRPM8 [20], [21], [22], [23], [24]. Similarly, while BCTC inhibits TRPM8-mediated Ca^2+^ influx, [24], this compound also functions as a TRPA1 agonist [25]. Likewise, the anti-fungal medication clotrimazole has strong TRPM8 antagonistic activity, but also robustly activates TRPV1 and TRPA1, actions consistent with the commonly reported side effects of irritation and burning [26]. SKF96365, a non-specific blocker of several types of calcium channels, receptor-operated channels, and inwardly rectifying potassium channels [27], [28], also inhibits TRPM8 in vitro [29]. Certain tryptamine derivatives that are ligands for 5-benzyloxytryptamine receptors also act as TRPM8 antagonists [30]. Lastly, ethanol, at concentrations of 1–3%, inhibits TRPM8 channel function by disrupting interactions with the membrane phospholipid phosphatidylinositol-4,5-bisphosphate (PIP_2_) [17], [31], an obligate molecule for TRPM8 channel function [32], [33]. Together, these chemicals present a range of pharmacological tools to regulate TRPM8 function, yet each of these compounds has off-target effects, thus complicating their utility in the investigation of the role of TRPM8 as a drug target.

**Figure 1:**
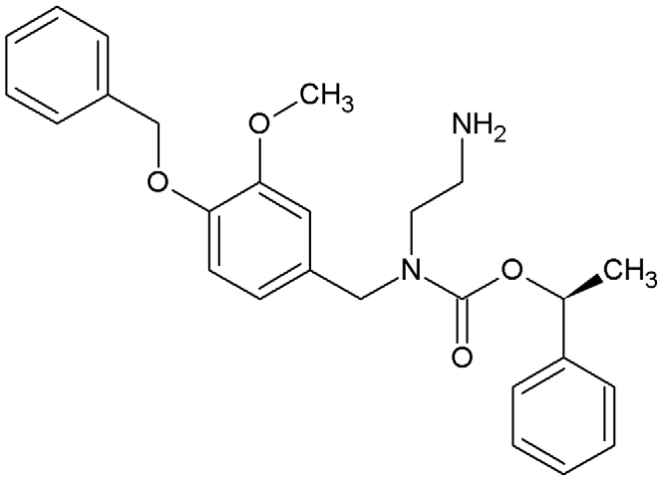
Structure of PBMC. 1-phenylethyl-4-(benzyloxy)-3-methoxybenzyl(2-aminoethyl)carbamate.

The goal of this study was to determine the selectivity and potency of a candidate TRPM8 antagonist 1-phenylethyl-4-(benzyloxy)-3-methoxybenzyl(2-aminoethyl)carbamate (Figure 1; abbreviated PBMC) in inhibiting TRPM8 at the levels of *in vitro* channel function and *in vivo* behavior under both normal and pathological conditions. Our results show that PBMC is a suitable structural template for formulations of specific and highly potent TRPM8 antagonists. Moreover, blockade of TRPM8 disrupted thermoregulation and normal thermosensation as well as attenuated injury-evoked painful cold hypersensitivity, further establishing a role for TRPM8 in these physiological processes.

## Results

### PBMC selectively blocks TRPM8 activity

**Figure 2:**
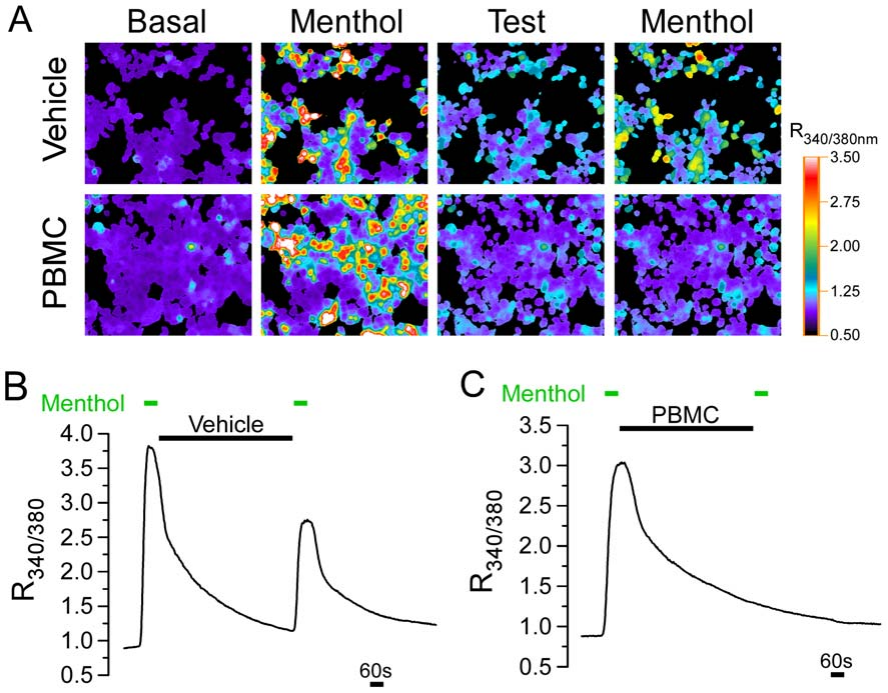
PBMC inhibits menthol-evoked TRPM8 responses. **A**) Representative images of HEK293T cells expressing mTRPM8. Pseudocolored images of the 340/380 nm (excitation) Fura-2 ratio (R_340/380_) show low basal Ca^2+^ before application of 200 µM menthol, which evoked a robust increase in intracellular Ca^2+^. A second application of menthol resulted in a second increase in intracellular Ca^2+^ after a ten minute treatment with vehicle (top row) but not after treatment with 25 nM PBMC (bottom row). **B**) Average changes in the Fura-2 ratio of vehicle-washed menthol-responding cells show that the second menthol pulse resulted in a robust calcium influx, albeit to a smaller degree than that of the first pulse. **C**) Average changes in the Fura-2 ratio of cells perfused with PBMC show that the drug abolished the second calcium increase.

**Figure 3:**
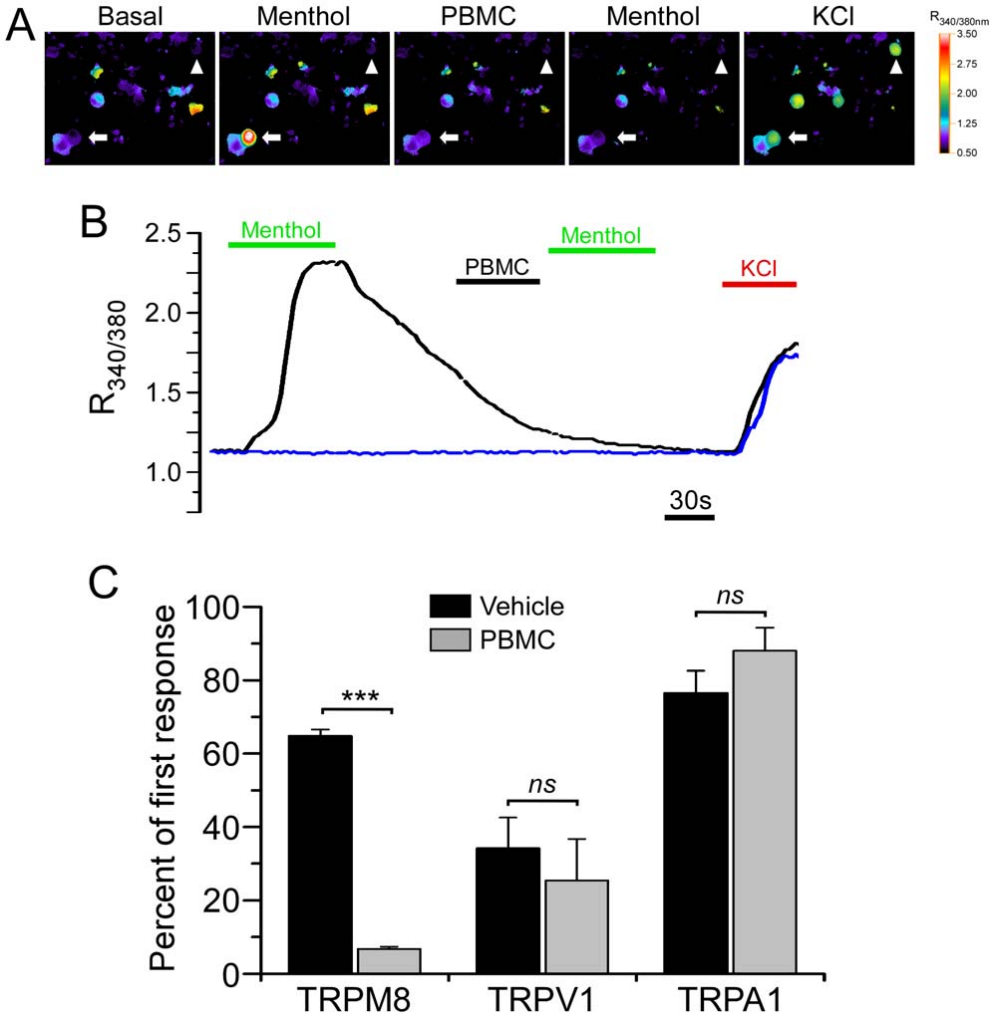
PBMC shows selectivity for TRPM8. **A**) Representative pseudocolor images (n = 4) of the Fura-2 ratio in cultured TG neurons. In this field a single cell is robustly activated by 200 µM menthol (arrow), but after treatment with PBMC (50 nM) a subsequent menthol application was ineffective. **B**) Ratio values of the cells shown in **A** (black trace: arrow; blue trace: arrowhead). Note that PBMC alone did not alter intracellular Ca^2+^ in menthol-insensitive, K^+^-sensitive neurons (arrowhead). **C**) Average peak ratio values of the second menthol response presented as a percentage of the first response compared for vehicle- (black bars; 65.0±2.0) and PBMC- (grey bars; 7.0±1.0) treated cells. 25 nM PBMC significantly inhibited menthol responses in HEK293T cells transfected with TRPM8 as compared with vehicle controls (Student's t-test, ***p<0.001). However, the drug did not affect capsaicin responses in TRPV1-transfected cells or AITC responses in TRPA1-transfected cells (Student's t-test, n.s. p>0.05).

We first tested the effects of PBMC on menthol-induced responses in heterologous cells expressing TRPM8 channels using calcium microfluorimetry [32]. In HEK293T cells transiently transfected with the mouse orthologue of TRPM8 (mTRPM8), brief and repeated exposure to 200 µM menthol evoked a robust increase in intracellular calcium, measured as a change in the Fura-2 fluorescence signal ratio (Figure 2A,B). Calcium levels returned to baseline over the course of ten minutes, and due to channel adaptation [32], the second menthol response was reduced in these assays, but still robustly increased intracellular Ca^2+^. To test the ability of the candidate compound to block TRPM8 activation, we perfused PBMC (25 nM) or vehicle between the first and second applications of menthol, observing complete abolishment of menthol-evoked Ca^2+^ responses at this concentration (Figure 2A,C). Data from several independent experiments showed that the average second response was 65.0±2.0% of the first response when vehicle was applied to the bath, compared to 7.0±1.0% with 25 nM PBMC (Figure 3C; n = 124 cells for vehicle, n = 108 cells for PBMC; Student's t-test, p<0.001).

Next we determined whether PBMC blocks TRPM8 activity in native cells. Mouse trigeminal ganglion (TG) neurons were enzymatically dispersed as described [32], and changes in intracellular Ca^2+^ were monitored as previously. Transient addition of 200 µM menthol evoked a robust increase in the Fura-2 ratio in a small fraction of cells (Figure 3A; arrow), whereas responses to a second application were abolished by preincubation with PBMC (Figure 3A,B; 50 nM). Of note, PBMC alone did not produce any change in intracellular Ca^2+^, nor did it prevent depolarization-induced Ca^2+^ increases evoked by addition of 50 mM KCl to the perfusate. These results show that PBMC also blocks TRPM8 activity in native cells and has no appreciable effects on intracellular Ca^2+^ transients evoked by K^+^-induced depolarization.

As stated above, all known TRPM8 antagonists have effects on other ionic mechanisms, as either antagonists or agonists, including the somatosensory-relevant channels TRPV1 and TRPA1. Thus, to further test the selectivity of PBMC for TRPM8, we performed similar calcium imaging experiments with heterologous cells expressing TRPV1 or TRPA1. Cells transfected with rTRPV1 were subjected to two challenges with the TRPV1-specific agonist capsaicin (1 µM), separated by incubation with either vehicle or PBMC (25 nM). The averaged second responses were 34.0±8.0% for vehicle and 25.0±11.0% for PBMC (Figure 3C; n = 84 cells for vehicle, n = 84 cells for PBMC; Student's t-test, p>0.05). Similarly, when cells transfected with rTRPA1 were challenged twice with 10 µM allyl isothiocyanate (AITC), the averaged second responses were 77.0±6.0% for vehicle and 88.0±6.0% for PBMC (25 nM; Figure 3C; n = 187 cells for vehicle, n = 87 cells for PBMC; Student's t-test, p>0.05). Furthermore, we did not find any evidence of PBMC acting as an agonist for these channels (not shown). Thus, PBMC shows selective inhibition of heterologously expressed TRPM8 over TRPV1 and TRPA1.

**Figure 4:**
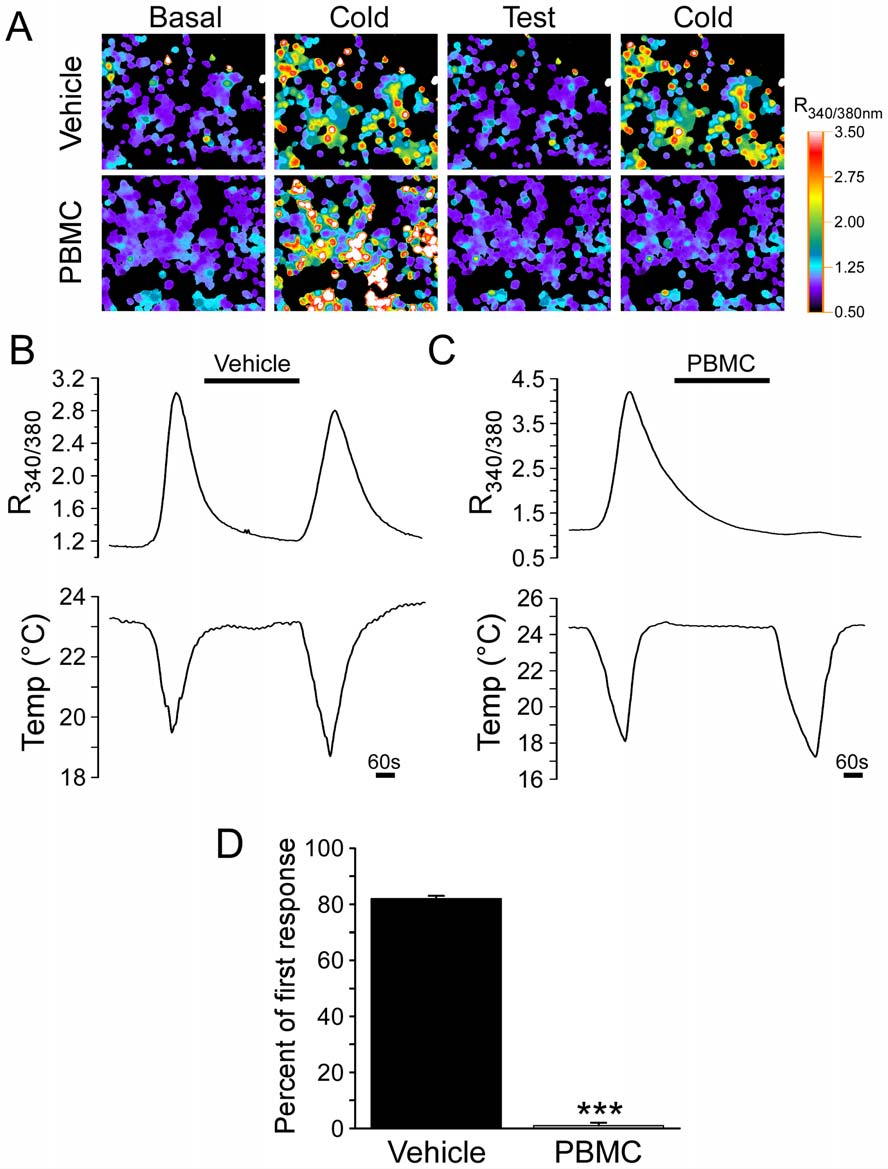
PBMC inhibits cold-evoked TRPM8 responses. **A**) Representative images of HEK293T cells expressing mTRPM8. Pseudocolored images of the 340/380 nm Fura-2 ratio (R_340/380_) show low basal Ca^2+^ before cooling the bath solution, which evoked a robust increase in intracellular Ca^2+^. After treating the cells for five minutes with vehicle (top row) or 25 nM PBMC (bottom row), cells again displayed low basal Ca^2+^ levels. A second cooling of the bath resulted in a calcium increase in vehicle, but not PBMC-treated cells. **B**) Average changes in the R_340/380_ of vehicle-washed cold-responding cells show that, under these conditions, the second cold pulse resulted in a robust influx of calcium into the intracellular space which was only slightly smaller than that seen for the first cold pulse. **C**) Average changes in the R_340/380_ of cells washed with PBMC show that the drug abolished the second calcium influx. **D**) 25 nM PBMC significantly inhibited cold responses in HEK293T cells transfected with TRPM8 as compared with vehicle controls. Data are presented as the average value of the second response as a percentage of the first compared for vehicle- (black bars; 82.0±1.0) and PBMC- (grey bars; 0.4±0.4) treated cells (Student's t-test, ***p<0.001).

Next, we tested whether PBMC also attenuated cold-induced TRPM8 activity, again using calcium microfluorimetry. In HEK293T cells transiently transfected with mTRPM8, cooling of the bath solution (from ∼24 to 18°C) evoked a robust and reproducible increase in intracellular calcium that returned to baseline after the bath solution returned to room temperature (Figure 4A,B), similar to what we have reported previously [32]. Consecutive cooling ramps separated by a five-minute incubation with vehicle elicited calcium responses of similar magnitudes, with the second response averaging to 82.0±1.0% of the first cold challenge (Figure 4A,B,D; n = 70 cells). However, when 25 nM PBMC was applied between the cooling ramps, the second calcium response was negligible at a value of 1.0±1.0% of the first response (Figure 4A, C, D; n = 65 cells; Student's t-test, p<0.001 vehicle vs. PBMC). We therefore conclude that PBMC inhibits both chemical and thermal activation of TRPM8.

### PBMC inhibits TRPM8 channels in a dose-dependent manner

**Figure 5:**
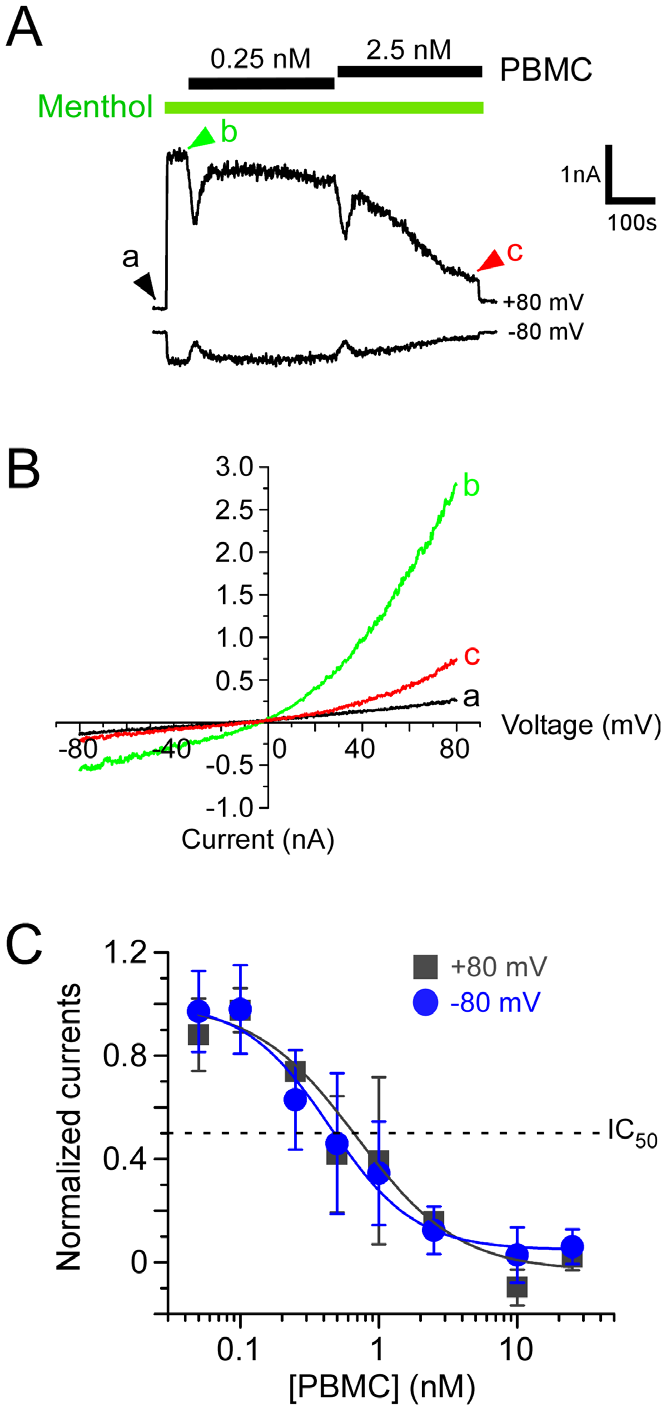
PBMC inhibits TRPM8 currents. **A**) Representative whole-cell voltage clamp recording from a mTRPM8-expressing HEK293T cell. Currents, at both positive and negative potentials were measured during a voltage ramp from −80 mV to +80 mV (1 V/s) and evoked with 500 µM menthol (in the absence of extracellular calcium, with 10 mM EGTA in the pipette), followed by the addition of 0.25 nM then 2.5 nM PBMC for five minutes per concentration. Menthol was present in the perfusate for the duration of the drug application (green bars) and a perfusion artifact was inserted to demarcate the solution change. **B**) Current-voltage relationships at the time points indicated in **A**. **C**) Normalized currents plotted against a range of PBMC concentrations. Reduction of TRPM8 currents by PBMC was dose-dependent at +80 mV (squares) and −80 mV (circles). The calculated IC_50_ values were 0.6 nM and 0.4 nM at positive and negative voltages, respectively (n = 6–8 cells per data point).

To further characterize the effect of PBMC on TRPM8 function, we turned to whole-cell voltage clamp recordings as a more direct method of observing the drug's actions on TRPM8 channels. In heterologous cells, we recorded menthol-evoked currents in calcium-free conditions (nominally Ca^2+^ free external solutions and 10 mM EGTA in the pipette) in order to exclude calcium-mediated adaption from our analysis [32], [33]. Under these experimental conditions, menthol-evoked TRPM8 currents are remarkably stable and show little to no rundown [1], [32], [33]. At both positive (+80 mV) and negative (−80 mV) membrane potentials, a saturating concentration of menthol (500 µM) evoked robust currents with strong outward rectification (Figure 5A,B). These responses were significantly reduced by PBMC at concentrations as low as 0.25 nM (Figure 5A), with nearly complete functional block of channel activity at 2.5 nM (Figure 5A,B). Dose-response relationships were performed and by fitting the data with a sigmoidal Hill equation we observed an IC_50_ of 0.6 nM at +80 mV and 0.4 nM at −80 mV (Figure 4C). The Hill coefficient, *n*, was measured to be 1.1 and 1.4 at +80 mV and −80 mV, respectively, suggesting a single binding site. This makes PBMC the most potent TRPM8 antagonist reported to date [16]. Moreover, the effects of PBMC were irreversible with no recovery of menthol-evoked currents even after a twenty minute washout (data not shown).

### PBMC shifts the voltage-dependence of TRPM8 channels

**Figure 6:**
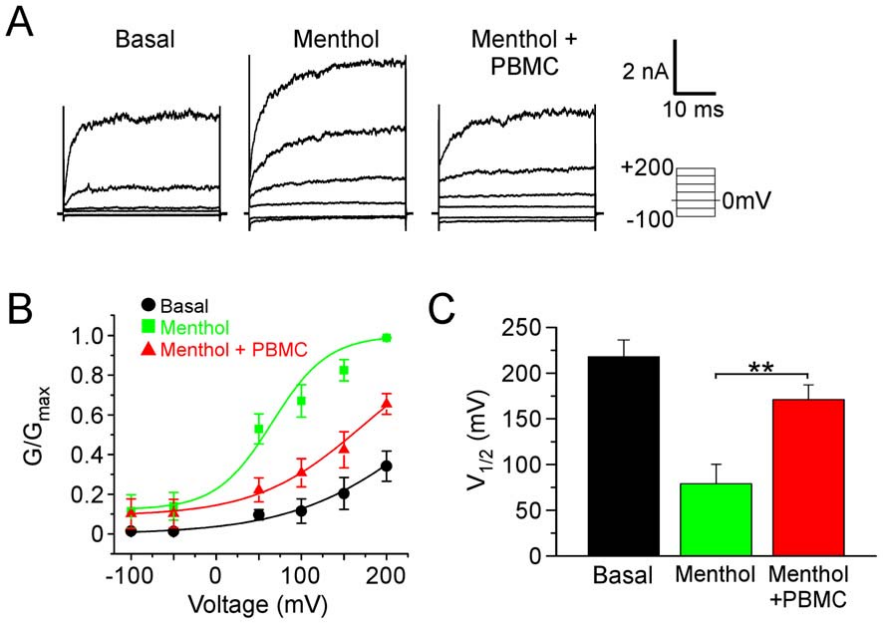
PBMC shifts the voltage dependence of TRPM8 channel gating. **A**) Representative whole-cell TRPM8 current traces in response to the indicated voltage step protocol. Traces show activity before and after application of 1 mM menthol and after application of 0.5 nM PBMC while still in the presence of 1 mM menthol. **B**) Steady-state activation curves under basal, menthol, and menthol + PBMC conditions. The normalized conductance (G/G_max_) was plotted against voltage and the addition of PBMC resulted in a reduction of the normalized conductance. Lines represent Boltzmann functions fitted to the data (n = 6–8 cells). **C**) Average voltages (mV) of half-maximal normalized conductance (V_1/2_) obtained from the Boltzmann functions in B. The addition of PBMC with menthol shifted the V_1/2_ from 79.1±21.1 mV to 171.2±16.0 mV (Student's t-test, **p<0.01), towards the baseline value of 218.2±18.2 mV.

TRPM8 gating is weakly voltage-dependent in that agonists such as menthol and cold shift channel activation properties towards more negative membrane potentials, thereby facilitating channel opening at physiological voltages [34], [35]. Previously we found that calcium- and PIP_2_-dependent adaptation also correlates with a shift in TRPM8 voltage-dependence, but towards more positive membrane voltages, thereby reducing channel gating [32]. Therefore we asked whether PBMC's effects on menthol-evoked TRPM8 conductances produce a similar shift in the voltage dependence of the channel. In heterologous cells expressing mTRPM8, steady-state menthol-evoked currents were recorded at 23°C during voltage steps (−100 to +200 mV) from a holding potential of 0 mV under basal conditions, in the presence of 1 mM menthol, or with 1 mM menthol and a near-IC_50_ concentration of PBMC (0.5 nM; Figure 6A). Normalized TRPM8 conductances for each cell, referred to as G/G_max_, were plotted for the given voltages under the three experimental conditions. We found that application of menthol alone shifted the activation curve towards negative membrane potentials, as reported previously [32], [34], [35]. However, in the presence of PBMC, the menthol-induced normalized conductance shifted back towards the basal state and more positive membrane potentials (Figure 6B). The conductance-to-voltage relationship was fitted with a Boltzmann function and we calculated the half-maximal activation voltage (V_1/2_) under each condition. The average basal (23°C) V_1/2_ was 218.2±18.2 mV, with the addition of menthol shifting this value to 79.1±21.1 mV (Figure 6C), data consistent with previous reports [32], [34]. When 0.5 nM PBMC was added to the bath with menthol, this shifted the average V_1/2_ to 171.2±16.0 mV (Student's t-test, p<0.01 menthol vs menthol + PBMC). These results suggest that PBMC antagonizes TRPM8 activity by shifting the voltage-dependence of channel gating toward more positive voltages, thereby partially reversing the effects of agonist activation.

### PBMC affects body temperature in a dose-dependent manner

Our in vitro data show that PBMC is a profoundly potent TRPM8 antagonist with sub-nanomolar affinity. Therefore we next determined if this compound is equally effective in blocking channel function *in vivo*. It has been previously reported that TRP channel antagonists can affect thermoregulatory processes, most notably the undesired hyperthermic effect seen with TRPV1 antagonism [13]. Similarly, the potent TRPM8 agonist icilin is well known to produce an intense behavioral response in rodents that is manifested as shivering, “wet-dog” shaking and also results in an increase in core body temperature in rats [11], [36], [37]. To date, all known *in vivo* effects of icilin are dependent on TRPM8 [5], [6], yet genetic evidence demonstrating that icilin-induced hyperthermia is TRPM8-dependent is yet to be established.

**Figure 7:**
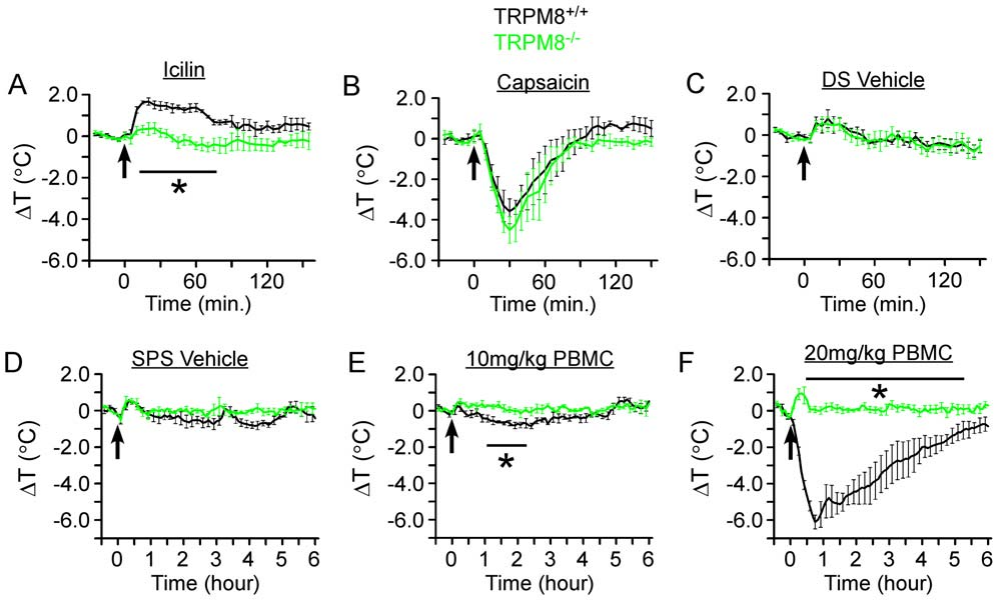
PBMC affects thermoregulation in a dose-dependent manner. **A**) Injection of 10 mg/kg icilin resulted in an average increase in core body temperature of 1.6°C as measured by thermal telemetry. This hyperthermic response to icilin was not present in TRPM8^-/-^ animals, which only exhibited a mild (<0.5°C) and transient (<30 minutes) increase, similar to that observed with vehicle (**C**). **B**) Injection of 1 mg/kg capsaicin resulted in a robust hypothermic response (∼4°C drop) in wildtype and TRPM8^-/-^ animals. **C**) Injection of vehicle (20% DMSO/80% saline (DS)) resulted in no change in core body temperature in either genotype beyond a small spike in body temperature within 30 minutes of injection. **D**) Intraperitoneal injection of warmed 10% Solutol/20% PEG-200/saline (SPS) vehicle resulted in no changes in core body temperature besides the injection spike in either genotype. **E**) Injection of warmed 10 mg/kg PBMC resulted in a subtle hypothermic effect (<1°C drop) in wildtype mice which resolved within three hours of injection, whereas TRPM8^-/-^ mice remained unaffected. **F**) Injection of warmed 20 mg/kg PBMC resulted in a profound hypothermic response (>6°C drop within 45 minutes) in wildtype animals, which did not occur in TRPM8^-/-^ mice. Arrows indicate injection time. Some error bars were omitted for clarity and all data are from 4–8 animals. Bars denote data that was statistically different (* p<0.05) between wildtype and TRPM8^-/-^ mice.

Therefore, we first examined the role of TRPM8 activation in thermoregulatory responses by subcutaneously injecting 10 mg/kg icilin into wildtype and TRPM8-knockout mice (TRPM8^-/-^) implanted with thermal telemeters [3], [6]. Consistent with data in rats [36], we observed a pronounced hyperthermic effect of 1.6°C on average in wildtype mice, which resolved within 90 minutes (Figure 7A; n = 4). However, this hyperthermic response was absent in TRPM8^-/-^ mice, with only a small injection-related artifact observed that was similar to vehicle injections (Figure 7A, C; n = 4). When we administered 1 mg/kg capsaicin *s.c.* to wildtype and TRPM8^-/-^ mice we found a profound and transient hypothermic effect of around 4°C that was similar in both genotypes, indicating that the TRPM8^-/-^ mice were still able to mount a chemically-induced thermoregulatory response (Figure 7B). Injection of the DMSO/saline (DS) vehicle *s.c.* induced only a brief increase in body temperature of around 0.5°C which peaked within 30 minutes post-injection in both genotypes (Figure 7C; n = 4 each genotype).

We next determined if PBMC antagonism of TRPM8 alters thermoregulatory responses in a likewise, yet reversed, manner. However, we found that subcutaneous injections of the required vehicle for PBMC (10% Solutol/20%PEG-200/saline (SPS)) resulted in intense grooming and scratching at the site of injection in both wildtype and TRPM8^-/-^ mice. Since stress is known to influence thermoregulation [38], we therefore switched to intraperitoneal injections of solutions warmed to 37°C immediately before injection and administered as far away from the telemeter implantation site as possible. This approach resulted in no obvious adverse effects associated with intraperitoneal vehicle injections (Figure 7D). Next, we tested a range of PBMC doses (2, 10, 20 mg/kg), finding no effect with 2 mg/kg (identical to vehicle, data not shown) and a small, but significant drop in core body temperature with 10 mg/kg which peaked at 0.8°C below baseline by two hours post-injection (Figure 7E; n = 4; p<0.05). Strikingly, at 20 mg/kg, we observed a dramatic and severe hypothermic effect of more than 6°C, with a drop in core body temperature to below 30°C in one instance (Figure 7F; n = 4). The drop in core body temperature of more than two degrees lasted at least four hours on average. Importantly, TRPM8^-/-^ mice showed no fluctuations in core temperature besides the transient injection artifact at all doses (n = 4 each dose). These data show that blockade of TRPM8 activity at high PBMC doses significantly alters thermoregulation, providing pharmacological evidence that, like TRPV1 [12], TRPM8 is involved in the maintenance of core body temperature.

### PBMC affects acute cold thermosensation

**Figure 8:**
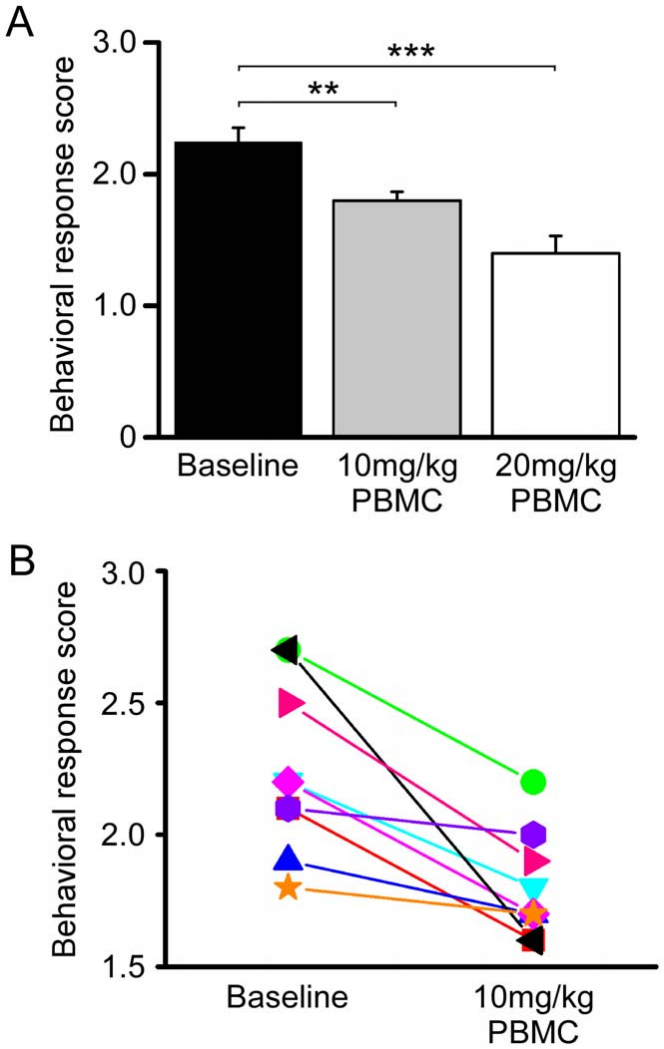
PBMC reduces acute cold-evoked behavioral responses. **A**) In the acetone evaporative cooling assay, wildtype mice exhibited an average response score of 2.2±0.1, while treatment with 10 mg/kg PBMC reduced this score to 1.8±0.1 (Student's t-test, **p<0.01 vs. baseline) and treatment with 20 mg/kg PBMC reduced it further to 1.4±0.1 (Student's t-test, ***p<0.001 vs. baseline). **B**) Every mouse given 10 mg/kg PBMC showed a decrease in acetone response scores ranging from 0.1 (orange stars, violet hexagons) to 1.1 (black triangle).

We and others have previously reported that TRPM8 is required for behavioral responses to cooling over a broad range of cold temperatures [3], [4], [5], [6]. To test whether pharmacological blockade of TRPM8 channels by PBMC affects normal thermosensation, we gave mice intraperitoneal injections of 10 or 20 mg/kg PBMC and assayed cold thermosensation at one hour post-injection using the evaporative cooling assay [3], [4]. When acetone is applied to the mouse's hindpaw, behavioral responses can be scored according to the magnitude of the response [4]. Here, the scores range from zero to five, with a zero score indicating no response and a five the most severe response, which we observed to be prolonged guarding of the hindpaw. When wildtype mice were subjected to this test, the resulting average score for both paws was 2.2±0.1 (Figure 8A; n = 9 mice). However, when these animals were given 10 or 20 mg/kg PBMC, their scores decreased to 1.8±0.1 or 1.4±0.1, respectively, with every mouse given the lower dose exhibiting a decrease in response score (Figure 8A,B; Student's t-test vs. baseline, p<0.01 for 10 mg/kg and p<0.001 for 20 mg/kg). As the higher PBMC concentration lead to a significant drop in core body temperature during the test period (Figure 7F), we cannot exclude the possibility that the observed behaviors are affected by the hypothermia associated with this dose. Nonetheless, there were significantly reduced cold behaviors with 10 mg/kg PBMC, a dose that did not produce a change in core temperature beyond that observed with circadian rhythms (35.3–38.0°C; Figure 7E and data not shown), suggesting that the drug altered acute cold sensation.

### PBMC attenuates injury-induced cold hypersensitivity

**Figure 9:**
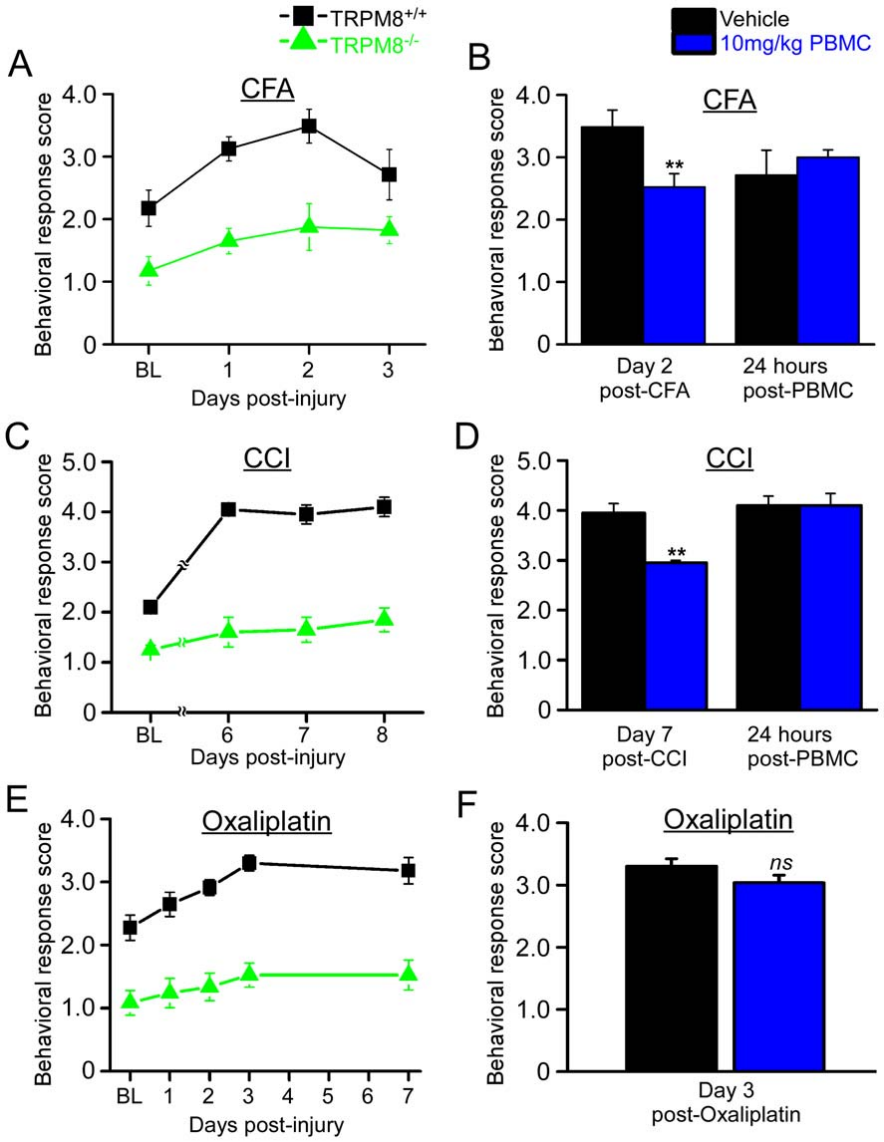
PBMC reduces cold hypersensitivity in CFA and CCI but not oxaliplatin pain models. **A**) In the CFA model of inflammatory pain, wildtype animals showed an increase in behavioral response scores which peaked at 3.5±0.3 at two days post-injury, whereas scores for TRPM8^-/-^ mice did not change significantly from the baseline (BL) of 1.9±0.4 (ANOVA, p>0.05). **B**) PBMC (10 mg/kg) administered to wildtype mice on day two post-CFA-injection resulted in a significant decrease in scores to 2.5±0.2 (Student's t-test, **p<0.01). The effect of the drug was gone within 24 hours, with PBMC-treated animals average score of 3.0±0.1, which was not significantly different from the 2.7±0.4 score of vehicle-treated animals (Student's t-test, p>0.05). **C)** In the CCI model of neuropathic pain, wildtype animals exhibited a response score of 4.1±0.1 by day six post-injury, which remained constant through day eight. TRPM8^-/-^ mice exhibited no significant increase in responses over baseline, with scores reaching 1.7±0.3 by day six post-injury (ANOVA, p>0.05). **D)** Treatment of CCI-wildtype animals with 10 mg/kg PBMC resulted in a decrease in score on day seven post-injury to 3.0±0.1, which was significantly lower than 4.0±0.2 in vehicle-treated animals (Student's t-test, **p<0.01). Scores of PBMC-injected animals increased to 4.1±0.2 by 24 hours post-treatment, which was the same score for vehicle-treated animals. **E)** In the oxaliplatin-induced model of neuropathic pain, wildtype animals showed a peak score of 3.3±0.1 by day three, which remained constant through day seven at 3.2±0.2. TRPM8^-/-^ animals showed no significant changes in score over the seven day period (ANOVA, p>0.05). **F)** Wildtype animals treated on day three post-injury with 10 mg/kg showed a slight reduction (3.0±0.1) in acetone score as compared to vehicle-treated animals (3.3±0.1) although this decrease was not statistically significant (Student's t-test, p = 0.08).

In addition to acute cold sensitivity, TRPM8 has also been reported to be necessary for cold hypersensitivity in both inflammatory and neuropathic pain models [4], [9]. Therefore, we next sought to determine if PBMC could alleviate these symptoms in wildtype animals. First, to confirm that TRPM8 is indeed required for inflammatory cold hypersensitivity we used unilateral intraplantar injections of complete Freund's adjuvant (CFA) into the hindpaws of wildtype and TRPM8^-/-^ mice. When CFA was injected into one hindpaw in wildtype mice, the acetone response scores for that paw increased from 2.2±0.3 before the injection to a peak of 3.5±0.3 by two days post-injury (Figure 9A; n = 8; ANOVA, p<0.05). No changes were observed in the un-injected contralateral paw (not shown). However, in TRPM8^-/-^ mice, CFA injection did not significantly change these responses beyond the reduced behaviors we already observed in TRPM8^-/-^ animals at baseline (Figure 9A; 1.9±0.4; n = 8; ANOVA, p>0.05). These data reaffirm the previous report that CFA-induced cold hypersensitivity is TRPM8-dependent [4].

Similarly, neuropathic pain induced by the chronic constriction injury (CCI) of the sciatic nerve induces symptoms of cold hypersensitivity which have also been reported to be TRPM8-dependent [4]. We confirmed these results, finding that by day six post-injury, the acetone scores of wildtype mice were 4.1±0.1 (up from 2.1±0.1 at baseline), which remained constant over the following two days (Figure 9C; n = 4). TRPM8^-/-^ mice exhibited a score of 1.6±0.3 by day six post-injury, which was not significantly different from the baseline value of 1.3±0.1 and did not significantly increase over the next two days (Figure 9C; n = 4; ANOVA, p>0.05). As with the inflammatory model, these data reaffirm the role of TRPM8 in CCI-evoked cold hypersensitivity [4].

Next we tested whether PBMC could reduce cold hypersensitivity in these two pain models. For CFA-induced inflammation, when 10 mg/kg PBMC was injected on the peak response day (day two post-injury), we observed a response score of 2.5±0.2 (n = 8) one hour after drug administration, which was significantly lower than the vehicle control group (Figure 9B; Student's t-test, **p<0.01). The effect of PBMC wore off within 24 hours, when acetone responses scores increased to 3.0±0.1, values not significantly different from the vehicle control group (2.7±0.4; Student's t-test, p>0.05). Similarly, in the CCI model, when 10 mg/kg PBMC was administered to injured wildtype mice on day seven post-injury, the behavioral response scores dropped to 3.0±0.1 one hour after the injection, a significant decrease when compared to vehicle-treated animals (Figure 9D; n = 4; Student's t-test, p<0.01). As for CFA, this amelioration of cold hypersensitivity was transient with animals returning to the sensitized state 24 hours later (Figure 9D). Thus PBMC is effective in diminishing symptoms of cold hypersensitivity in these two models of inflammatory and neuropathic pain.

Finally, we tested the effect of PBMC on a systemic neuropathic injury model. The platinum-based chemotherapeutic drug oxaliplatin is known to induce significant cold hypersensitivity which has been attributed to TRPM8 [9], [39], [40]. Animals injected with oxaliplatin developed a heightened response to acetone application that increased from 2.3±0.2 at baseline to 3.3±0.1 by day three post-injection and remained constant through day seven post-injury (Figure 9E; n = 8). This increase was absent in TRPM8^-/-^ mice injected with oxaliplatin (1.1±0.2 at baseline; 1.5±0.2 by day three post-injury; n = 8; ANOVA, p>0.05), thus confirming that the channel is required for oxaliplatin-induced cold hypersensitivity. However, unlike the CFA and CCI models, 10 mg/kg PBMC did not significantly attenuate cold hypersensitivity when administered on day three post–injection, with scores only decreasing to 3.0±0.1 as compared to 3.3±0.1 for vehicle-treated animals (Figure 9F; n = 8 each treatment; Student's t-test, p = 0.08). Therefore, at a dose of 10 mg/kg, PBMC is effective at attenuating symptoms of cold hypersensitivity in the CFA model of inflammatory pain and the CCI model of neuropathic pain, but not in the systemic oxaliplatin-induced neuropathic pain model. We did not test higher doses due to the significant effects on thermoregulation (Figure 7F) which would likely complicate interpretation of these results.

## Discussion

Here we show that PBMC is a robust and selective TRPM8 antagonist. *In vitro*, PBMC is the most potent TRPM8 antagonist reported to date and inhibits channel activation to both chemical and thermal stimuli. Using calcium microfluorimetry and whole-cell electrophysiology, we found that PBMC reduced TRPM8 activity in a dose-dependent manner. Indeed, we observed an IC_50_ concentration of less than 1 nM, a dosage approximately 100-fold lower than the most potent TRPM8 antagonist reported to date, CTPC [16], [17]. Thus, the two-orders-of-magnitude higher affinity of PBMC makes this compound a more amenable reagent in the study of TRPM8 channel function.

Importantly, and unlike other TRPM8 antagonists, we did not observe any cross reactivity with either TRPV1 or TRPA1, suggesting that PBMC is selective for TRPM8. However, these observations are not all inclusive of other cellular mechanisms, but application of PBMC to cultured TG neurons did not lead to any noticeable changes in cellular excitability, suggesting that PBMC does not have any appreciable off-target effects at the level of cultured sensory neurons. We found that PBMC exerts its antagonistic effect on TRPM8 by shifting the voltage-dependence of TRPM8 gating. This particular result, consistent with previous reports from our lab and others, suggests that many (if not all types) of functional regulation of TRPM8—whether by agonist, antagonist, or adaptive mechanisms—involves changes in voltage-dependent gating [32], [34], [35], [41].

Emerging evidence suggests that TRPM8 plays a role in thermoregulation, both with the stimulation of skin afferents with chemical agonists [36], [42], [43] or cooling [11]. Here, we have confirmed that icilin, a chemical TRPM8 agonist more potent than menthol [16], [37] can also induce an increase in body temperature [36], an effect that is TRPM8-dependent [5], [6], despite reports that icilin can also activate TRPA1 *in vitro* [44]. Even though TRPM8^-/-^ mice do not respond to icilin, these animals retain the ability to mount a chemically-induced thermoregulatory response as we observed an identical effect in both wildtype and TRPM8^-/-^ mice in response to the TRPV1-agonist capsaicin. Therefore it appears that TRPM8-expressing afferents have the ability to affect thermoregulatory responses to both chemical and thermal stimuli, although the exact neurological mechanism remains to be explored.

Due to this evidence and recent reports of TRPV1 antagonists having undesired thermoregulatory effects [12], [13], we were concerned that a TRPM8 antagonist would also affect thermoregulation. Indeed, when we administered PBMC at a dose of 20 mg/kg, we observed a profound hypothermic effect, with one mouse reaching body temperatures below the temperature range of the telemeter (<30°C), a temperature classified as deep hypothermia in humans [45]. The pharmacokinetics of PBMC are as yet unknown, yet the hypothermic effect observed here lasted around four hours on average, and in thermoregulatory and behavioral experiments the effects were gone by less than one day after administration. Interestingly, halving the dose (10 mg/kg) almost completely abolished the hypothermic response, with core body temperatures dropping less than one degree—a surprising change in effect for such a small reduction in dose. Indeed, while this drop in core temperature was significantly different than vehicle injected control or TRPM8^-/-^ mice, it was not significant when compared to normal circadian changes in body temperature we observed in these mice. Thus, we suggest that the slight change in core temperature observed at the 10 mg/kg dose did not participate in the ability of PBMC to block acute cold sensation, as well as reduce injury-induced cold hypersensitivity.

It has been shown extensively that TRPM8 is required for cold sensation, particularly in the evaporative cooling assay [3], [4], [5], [6]. When a small volume of acetone is applied to the hindpaw of a mouse, it quickly evaporates and cools the skin down to temperatures as low 14–18°C [4], which is near the loose boundary of the transition from innocuous cool to cold pain [46]. With 10 mg/kg PBMC, we observed a partial reduction in the normal acetone response score, demonstrating that by blocking TRPM8, this compound can alter cold thermosensation. These responses were further reduced with the highest concentration tested, 20 mg/kg, although the interpretation of these effects are complicated by the dramatic hypothermia produced at this dosage. It is important to note that the PBMC-treated scores did not drop to the level of TRPM8^-/-^ mice (Figure 9), indicating partial blockade of the channel at this dose. Interestingly, we observed individual differences in the amplitude of the score reduction with 10 mg/kg PBMC under normal conditions, which may suggest that, at this low dose, individual variations in physiology may affect drug action. However, due to the thermoregulatory effects described above, we were limited in the amount of drug we could administer to the mice without potentially confounding thermosensory responses.

TRPM8 has also been implicated in the painful cold hypersensitivity that is a distressing symptom of inflammatory and neuropathic conditions, as well as platinum-based chemotherapy drugs [4], [9]. It would therefore be greatly beneficial to both chronic pain and chemotherapy patients to have a drug which could control such symptoms. Thus we tested whether PBMC could reduce the behavioral responses to evaporative cooling in models of inflammatory and neuropathic pain. In the CFA model of inflammatory pain and the CCI model of neuropathic pain, we saw a reduction in the response scores of mice treated with 10 mg/kg PBMC. Interestingly, both of these reduced scores remained higher than those seen at baseline or with TRPM8^-/-^ mice, again suggesting that at this dose PBMC only partially blocked TRPM8 function *in vivo*. However, given that the aim of a good symptom-controlling drug would be to reduce the hypersensitivity to cold without abolishing normal thermosensation (e.g. numbness), this may not be a completely undesirable effect.

In contrast, when we examined oxaliplatin-treated animals given PBMC, we did not see a statistically significant reduction in response scores. It is puzzling that PBMC would be effective against one model of neuropathic pain (CCI) but not another. There are two probable explanations for this observation: First, it is possible that other mechanisms may also be involved in cold hypersensitivity in oxaliplatin-induced neuropathy and PBMC is ineffective against these mechanisms [47], although our and others' recent evidence suggests that TRPM8 plays a pivotal role in this pathology [9], [39]. Alternatively, it may be that the partial inhibition of TRPM8 we observe with PBMC prevents this compound from being effective in reducing the response scores in this pain model. Again, as we were constrained by the hypothermic side effect of a higher dose, we were unable to test if higher doses could provide some level of analgesia in oxaliplatin-induced cold hypersensitivity. Reformulation of the drug, if possible, may yield a compound that specifically targets sensory afferents without having the strong thermoregulatory effect observed here. Such a drug may bring much-needed relief to both chronic pain and chemotherapy patients experiencing symptoms of cold hypersensitivity. Nonetheless, our results show that PBMC is a potent and selective inhibitor of TRPM8, and that inhibition of this channel alters cold sensation, thermoregulation, and provides a modest level of relief in rodent models of injury-induced cold pain.

## Materials and Methods

### Animals

All mice used in this study were adults aged at least eight weeks. Wildtype and TRPM8-knockout mice [3], [6] were on the same C57Bl/6 genetic background, and all animals were provided standard mouse chow and water *ad libitum*. All procedures and tests were approved by the University of Southern California Institutional Animal Care and Use Committee (Protocol number: 10674; Approval date: 12/08/2010) and conducted in accordance with the recommendations of the International Association for the Study of Pain and the *NIH Guide for the Care and Use of Laboratory Animals*.

### Heterologous expression

Complementary DNA (cDNA) of mouse TRPM8 (gifts from A. Patapoutian), rat TRPV1, and rat TRPA1 (gifts from D. Julius) clones were transfected into the human embryonic kidney cell line 293-T (HEK293T) using TransIT-LT1 reagent (Mirus, Madison, WI) following the manufacturer's instructions. Cells were maintained in a 37°C incubator in 5% CO_2_ in DMEM containing 10% fetal bovine serum and 1% penicillin-streptomycin.

### Neuronal Cell Culture

Trigeminal ganglia were dissected from newborn mice and dissociated with 0.25% collagenase P (Roche Applied Science, Indianapolis, IN) in a solution of 50% DMEM (Dulbecco's Modification of Eagle's Medium with 4.5 g/L gluscose, L-glutamine and sodium pyruvate, Mediatech, Inc., Manassas, VA), and 50% F-12 (HAM F-12 Nutrient Mixture with L-glutamine, Invitrogen Corporation, Carlsbad, CA) for 30 minutes. The ganglia were then pelletted and resuspended in 0.05% trypsin at 37°C for 2 minutes, and triturated gently with a fire-polished Pasteur pipette in culture medium (DMEM/F-12 with 10% FBS and penicillin-streptomycin). Cells were then resuspended in culture medium with nerve growth factor 7S (Invitrogen Corporation, Carlsbad, CA) (100 ng/ml) and plated onto coverslips coated with Matrigel (BD Biosciences, Inc., San Jose, CA) (20 ul/ml). Cultures were examined 16–20 hours after plating.

### Calcium microfluorimetry

Intracellular Ca^2+^ was determined with the cell-permeable form of Fura-2 (Invitrogen, Carlsbad, CA) as described [1], [32] and pseudo-colored ratiometric images were captured on an Olympus IX70 fluorescent microscope with Sutter Lambda LS light source, Roper CoolSnap ES camera, and the MetaImaging Software suite. Solutions were gravity-fed through tubes connected to an 8-channel perfusion valve solution controller (Warner Instruments, Hamden, CT). Temperature readings were captured by a CL-100 Temperature Controller (Warner Instruments, Hamden, CT) through a PowerLab 8/30 digital-to-analog converter (ADInstruments, Colorado Springs, CO). Data are represented as the mean ± the standard error and statistical significance was determined using a Student's t-test.

### Mammalian cell electrophysiology

Voltage clamp recordings were performed as described [32]. Standard bath solution for whole-cell recordings contained (in mM): 136 NaCl, 5.4 KCl, 1 MgCl_2_, 1.8 CaCl_2_, 10 HEPES, 10 glucose, and 0.33 NaH_2_PO_4_ and adjusted to pH 7.4 with NaOH. Pipette solution contained (in mM): 140 CsCl, 10 EGTA, 2 MgATP, and 10 HEPES and adjusted to pH 7.4 with CsOH. Nominally Ca^2+^-free bath solutions contained (in mM): 136 NaCl, 5.4 KCl, 1 MgCl_2_, 10 HEPES, 10 glucose, and 0.33 NaH_2_PO_4_ and adjusted to pH 7.4 with NaOH. Recordings were performed using an Axopatch 200B amplifier and Digidata 1320 data acquisition board with pCLAMP 9.2 software (all Molecular Devices, Inc., Sunnyvale, CA). Solutions were gravity-fed through tubes connected to an 8-channel perfusion valve solution controller (Warner Instruments, Hamden, CT). Rapid solution exchange was performed as previously described [32]. Briefly, rapid bath solution exchange was achieved by placing the cell in a recording chamber (Warner Instruments, Hamden, CT) in front of a linear array of microperfusion pipes under computer control (Warner Instruments, Hamden, CT). All drugs used in our experiments were stored and handled following the manufacturer's instructions.

### Voltage-dependent gating data analysis

Data analysis was performed using Origin 8.1 (OriginLab Corporation, Northampton, MA). Steady-state activation curves were determined using previously described methods [32], [34], [35]. Briefly, to estimate maximal TRPM8 activity at a given voltage, we used a saturating dose of 1 mM menthol [1] at room temperature (23°C) to activate TRPM8 and measured currents at the end of each voltage step. We then calculated the conductance, *G*, at each data point, using the relation *g  =  I_ss_/V*, where *I_ss_* is the steady-state current at the end of a voltage step, and *V* is the voltage difference across the cell membrane. Because the conductance appears to saturate and reach a maximum, we calculated *G/G_max_* for each value, thus normalizing data so that comparisons could be made between cells. For simplicity, we assume for our calculations a two-state model of channel gating [34]. However, it should be noted that other reports have identified several additional channel states beyond the two originally described [32], [34], [35], [41], [48]. Therefore we fit the *G/G_max_* values with steady-state activation curves using a Boltzmann function of the form:
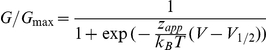
where *z_app_* is the experimentally determined gating charge, *k_B_* is the Boltzmann constant (1.38×10–23 J K^−1^) and *T* is the absolute temperature. The half-maximal conductance (*V_1/2_*) is estimated from these steady-state activation curves for each cell. Data are represented as the mean ± the standard error and statistical significance was determined using a Student's *t-*test.

### Pharmacological data analysis

Data analysis was performed using Origin 8.1 (OriginLab Corporation, Northampton, MA). Dose-response curves were fit with a Hill equation of the form
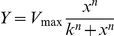
where *V_max_* is the maximum velocity of the reaction, *k* is the substrate constant, and *n* is the Hill coefficient. Data are represented as the mean ± the standard error.

### Thermal telemeter implantation and core temperature monitoring

Mice were implanted with G2 e-mitters (Mini Mitter, Bend, OR) according to the manufacturer's instructions. Briefly, under sterile conditions, mice were anesthetized with 4% isoflurane and maintained with 2% isoflurane in oxygen. The ventral surface was shaved and sterilized and a 2 cm incision was made in the skin, and a 1.5 cm incision made in the abdominal wall. A chemically-sterilized e-mitter was gently nestled amongst the small intestines with care not to compress any vital organs and anchored to the abdominal wall with 5–0 Vicryl sutures. The peritoneum was sutured, and the overlying skin closed with tissue adhesive. The animals were allowed to recover for 30 minutes in a warmed recovery cage before returning to their home cages. The animals were given 0.03 mg/kg buprenorphine 15 minutes prior to surgery and again every 12 hours post-surgery for a total of 48 hours. The animals' health and recovery were monitored by USC Department of Animal Resources staff. Animals were allowed to recover from surgery for at least one week to ensure the absence of infection and fever. On the day of experiments, animals were acclimated to the experiment room at least one hour prior to the commencement of temperature monitoring. The VitalView software package (Mini Mitter, Bend, OR) was used for automated temperature monitoring, with the temperature recording limits set to 40–30°C and the monitoring period set to every five minutes. Animals were provided standard mouse chow and water *ad libitum* during the testing period and allowed at least two days recovery between experiments. Baseline temperatures were calculated by averaging the temperature readings over the thirty minutes immediately prior to injection. The change in core temperature (ΔT) was calculated by subtracting the baseline temperature from the observed temperature. Care was taken to perform experiments at the same time of day so as to minimize circadian influences on temperature readings. Data are represented as the mean ± standard error and statistical significance was determined using a Student's t-test.

### Chemicals

Icilin (Tocris Bioscience, Ellisville, Missouri) was dissolved to a concentration of 24 mg/ml in DMSO and then diluted to 1 mg/ml in 20% DMSO/80% saline (DS vehicle) and administered at 10 mg/kg. A stock solution of 10 mg/ml capsaicin in ethanol was diluted to 0.1 mg/ml in the same vehicle and administered at a dose of 1 mg/kg. The vehicle was administered at the same volume (10 ml/kg) as both icilin and capsaicin. These solutions were administered subcutaneously to the dorsal surface of the animal. PBMC was a provided as a generous gift from Pfizer Inc. (Sandwich, Kent, U.K.) and dissolved in DMSO for cellular assays. For *in vivo* injections, the drug was suspended in a vehicle solution of 10% Solutol (Sigma-Aldrich, St. Louis, MO), 20% PEG-200 in normal saline (SPS vehicle) to a concentration of 2.5 mg/ml. PBMC and SPS vehicle solutions were administered either subcutaneously or intraperitoneally at doses of 2, 10, or 20 mg/kg, as outlined in the text. For behavioral testing, the animals were allowed to settle for one hour following PBMC injections.

### Evaporative cooling assay

The evaporative cooling assay was performed as follows: Mice were acclimated for fifteen minutes in an elevated, four-place chamber with a mesh floor. A syringe with a piece of rubber tubing attached to the end was filled with acetone and the plunger depressed so that a small drop of acetone formed at the top of the tubing. The syringe was raised to the mouse's hindpaw from below, depositing the acetone drop on the paw. Mice were tested four at a time with an inter-stimulation period of four minutes per mouse, alternating paws between stimulations. Responses were video recorded for later quantification by an observer blind to the experimental conditions. Behaviors were scored according to the magnitude of the response along the following scale: 0-no response; 1-brief lift, sniff, flick, or startle; 2-jumping, paw shaking; 3-multiple lifts, paw lick; 4-prolonged paw lifting, licking, shaking, or jumping; 5-paw guarding. The scale was designed so that the extreme values (0 and 5) occurred only rarely. Data are represented as the mean ± standard error. Statistical significance was assessed using either the paired or unpaired Student's t-test or one-way *ANOVA*, as appropriate.

### Pain models

Inflammatory injury was induced by unilateral intraplantar injection of 20 µl of complete Freund's adjuvant (CFA). The chronic constriction injury (CCI) model of neuropathic pain was induced as follows: Under sterile conditions, mice were anesthetized with 5% isoflurane and anesthesia maintained with 3% isoflurane in oxygen. The animal was positioned so that the right flank was accessible and the leg supported with a roll of gauze. The flank surface was closely shaved and sterilized and a 2 cm incision was made in the skin. The muscle was gently pried apart until the sciatic nerve was revealed. Three 6–0 chromic gut sutures were loosely tied around the nerve about 1 mm apart. The muscle was closed, and the skin was closed with tissue adhesive. The animals were allowed to recover for 30 minutes in a warmed recovery cage before returning to their home cages. The animals' health and recovery were monitored by USC Department of Animal Resources staff. Systemic neuropathic injury was induced by intraperitoneal injections of 3 mg/kg oxaliplatin (Sigma-Aldrich) dissolved in a 5% glucose/saline solution.
